# An Initial 5-Year Single-Center Experience of 365 Patients Undergoing the Video-Assisted Thoracoscopic Surgery for Nuss Procedure for Pectus Excavatum in Resource-Scare Setting

**DOI:** 10.3389/fsurg.2021.693562

**Published:** 2021-06-14

**Authors:** The-May Nguyen, Van-Thieu Le, Huu-Uoc Nguyen, Huu-Lu Pham, Hong-Son Duy Phung, Ngoc-Tu Vu, Viet-Anh Nguyen, Nam-Khanh Do, Kim-Duy Vu, Hoang-Long Vo, Quoc-Hung Doan

**Affiliations:** ^1^Department of Cardiovascular and Thoracic Surgery, Viet-Tiep Friendship Hospital, Hai Phong, Vietnam; ^2^Department of Cardiovascular and Thoracic Surgery, Viet Duc University Hospital, Hanoi, Vietnam; ^3^Department of Surgery, Hanoi Medical University, Hanoi, Vietnam; ^4^Department of Cardiovascular and Thoracic Surgery, Hanoi Medical University Hospital, Hanoi, Vietnam; ^5^Institute for Preventive Medicine and Public Health, Hanoi Medical University, Hanoi, Vietnam

**Keywords:** VATS, Nuss, congenital pectus excavatum, complications, thoracoscopic surgery

## Abstract

**Background:** Little is known about video-assisted thoracoscopic surgery in the Nuss procedure (VATS-NUSS) and its postoperative outcomes in the resource-scarce conditions in clinical practice such as Vietnam. Available evidence in the literature was mostly reported from large institutions in developed countries. Hence, this study was conducted to review our initial large single-center experience in the use of the VATS-NUSS for patients with pectus excavatum (PE) within 5 years.

**Methods:** Data from 365 consecutive PE patients between January 2015 and December 2019 who were surgically treated with VATS-NUSS were retrospectively analyzed.

**Results:** Of 365 patients, median age at operation was 15.61 ± 3.73 years (range = 5–27 years), most being child and adolescent. Three hundred nine patients (84.65%) were male. PE was commonly detected at puberty (*n* = 328, 89.9%). Postoperatively, early complications consisted of pneumothorax (*n* = 5, 1.37%), pleural bleeding/pleural fluid (*n* = 2, 0.55%), pleural hematoma (*n* = 1, 0.27%), pneumonia (*n* = 1, 0.27%), surgical wound infection (*n* = 1, 0.27%), incision fluid accumulation (*n* = 3, 0.82%), metal bar infection (*n* = 1, 0.27%), atelectasis (*n* = 3, 0.82%), and fever (*n* = 8, 2.19%). Late complications included surgical wound infection (*n* = 2, 0.55%), metal bar deviation (*n* = 5, 1.37%), metal bar allergy (*n* = 10, 2.74%), recurrent PE (*n* = 2, 0.55%), and persistent PE (*n* = 5, 1.37%). No deaths occurred. In 175 patients (47.95%) experiencing bar removal, mean operative time for bar removal was 34.09 ± 10.61 min, and the length of hospitalization following bar removal was 2.4 ± 1.34 days; the most frequent complication was pneumothorax (*n* = 19, 10.85%). One wound infection and one incision fluid accumulation happened following bar removal. Favorable midterm to long-term postoperative outcomes were achieved.

**Conclusions:** From the beginning of the Vietnamese surgeons' experience, VATS-NUSS application obtained favorable outcomes with minimizing the occurrence of serious intraoperative and postoperative complications. Current rare evidence enables to give a real picture in the application, modification, and development of VATS-NUSS in the countries having similar resource-scarce conditions.

## Introduction

With universal acceptance, the additional use of the video-assisted thoracoscopic surgery (VATS) technique in the Nuss procedure (NUSS) demonstrated an increased safety of the operation with decreased frequency of serious intraoperative and postoperative complications (1–6). Bufo et al. firstly, reported VATS use for the prevention of life-threatening intraoperative lesions of the mediastinum (7). The discussion is currently focused on the superiority of left, right, or bilateral thoracoscopic access; the necessity of CO_2_ insufflation; or the sternal elevation to improve the safety during surgical procedure (8–11). Although serious life-threatening complications after the NUSS are rare (12–14), VATS use during the operation may further reduce the perioperative risk. The available evidence in the literature was mostly reported from large institutions in developed countries, and no publications on the surgical outcomes and safety in the application of the VATS for NUSS were known in the resource-scarce conditions in clinical practice such as Vietnam.

To fill this knowledge gap, this study sought to review our large single-center experience in the use of the VATS technique in NUSS for the Vietnamese patients with pectus excavatum (PE) within 5 years. Our results might have some implications for establishing appropriate treatment strategies in the application, modification, and development of VATS-NUSS in the countries having similar resource-scarce conditions.

## Methods

### Patients

In this retrospective one-center study, we consecutively selected patients with the diagnosis of PE who underwent VATS for the NUSS procedure from January 2015 to December 2019. A total of 365 patients surgically treated for PE at our institution (Viet Duc University Hospital, Hanoi, Vietnam) were included in final analysis.

A comprehensive evaluation by complete history, physical examination, chest radiographs, electrocardiogram (ECG), pulmonary function test, echocardiogram, and computed tomography (CT) of the chest was performed for all patients. The demographic data, surgical data, early and late complications, length of hospital stay, and early, midterm, and long-term outcomes were also collected. All possible variables were finally included on the basis of clinical judgment, literature review, and availability in our hospital. The indications for surgical repair were two or more of the following criteria demonstrated by Dr. Nuss: (1) progression of the deformity, (2) exercise intolerance, (3) progressive chest pain or dyspnea, (4) restrictive ventilatory impairment, (5) Haller index >3.25, (6) previous failed Ravitch procedure, (7) cardiac compression, and (8) mitral valve prolapse. All procedures were performed with the NUSS, which included left, right, or bilateral VATS.

### Surgical Techniques

All patients had a thoracic epidural for intraoperative anesthesia and postoperative pain control. The patient was placed in the supine position after general anesthesia. The arms of patients were kept abducted approximately 70–80° in relation to the body.

In the determination of anatomic landmarks in surgery, we determined the concave area, the center of the concave area, the edges of the concave area, and the highest point of the concave edge on either side of the concave circumference. The metal bar was placed along a straight line that was formed from the center of the concave area and the two highest points of the concave edge. The measurements of the thorax and the bending of the metal bar (sternum lift) were then performed.

A 5-mm trocar was placed in the marked position on the left chest wall. This point was located in the sixth intercostal space on the midaxillary line. After pumping low pressure CO_2_ (5 mm Hg) into the pleural cavity to collapse the lungs, the camera with a 30-degree optic was taken to the left chest to observe the entire chest and mediastinum during surgery. Skin incision was made above or below the position where the metal rod was intended to be placed in an intercostal space. One 2-cm vertical skin incision was made in the midaxillary line each side. In required cases with two-bar insertion, also only a skin incision was made between the two intended positions to place the two metal bars. Subcutaneous or submuscular dissections were performed from the cutaneous incision to the highest edge of the PE.

**Figure 1:**
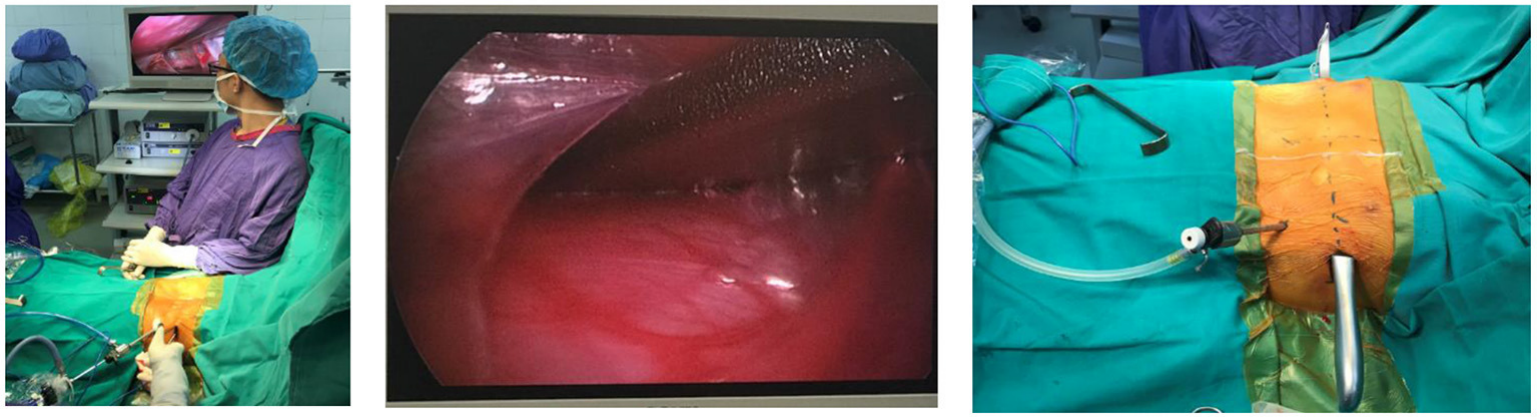
Process of the created tunnel through mediastinum under the control of thoracoscopy.

Then, we created a tunnel through the mediastinum under the control of thoracoscopy (Figure 1). The heart-shaped clamp was used to pierce the left pleural space at the highest edge of the pit, slowly went close to the anterior chest wall toward the mediastinum at the deepest point of the pit, and gradually separated the pericardium from the posterior sternum. The introducer reaches the posterior surface of the sternum in the left pleural cavity and further is moved through the mediastinal pleura in the retrosternal space, and then it went across the right pleural space and finally exits the chest through the right intercostal space. The ECG was continuously monitored during the process of created tunnel through the mediastinum.

**Figure 2:**
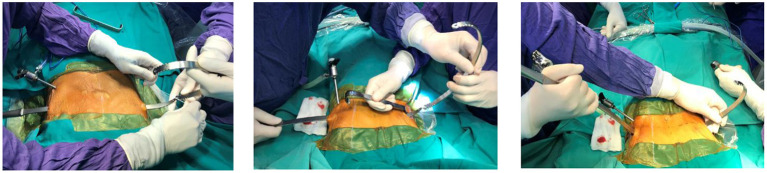
Metal bar was threaded through the left-to-right mediastinum.

**Figure 3:**
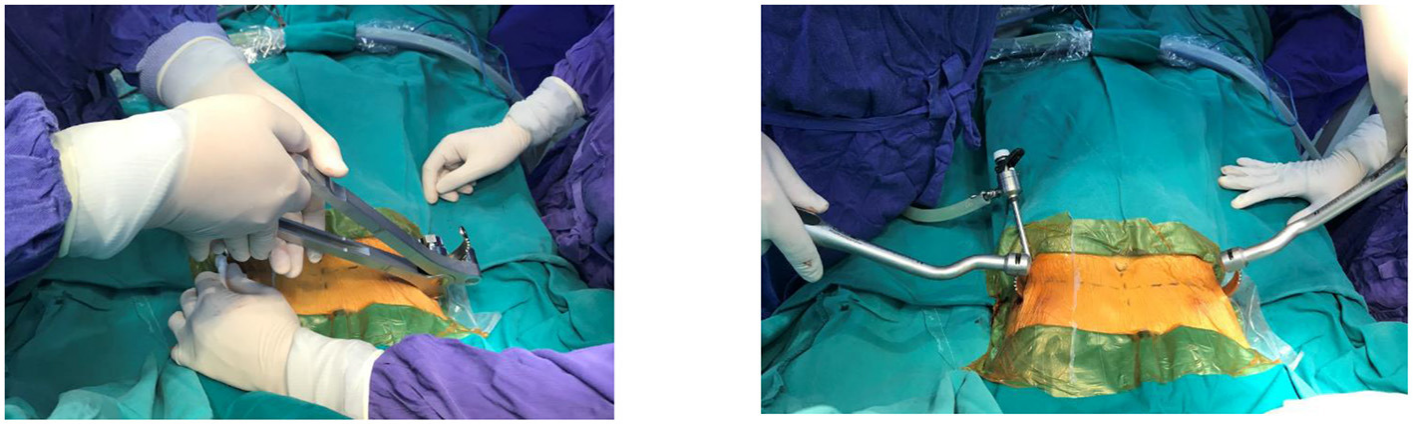
Bending and rotating the metal bar.

During the stage of reeving sternum lift bar, a Perlon thread was tied to the metal rod and then was tied to the introducer. Following the withdrawn introducer, the metal bar was threaded through the anterior mediastinum in a direction going from right to left under the control of the thoracoscopy (Figure 2). Then, the pectus bar was rotated to suit the patient's chest (Figure 3).

**Figure 4:**
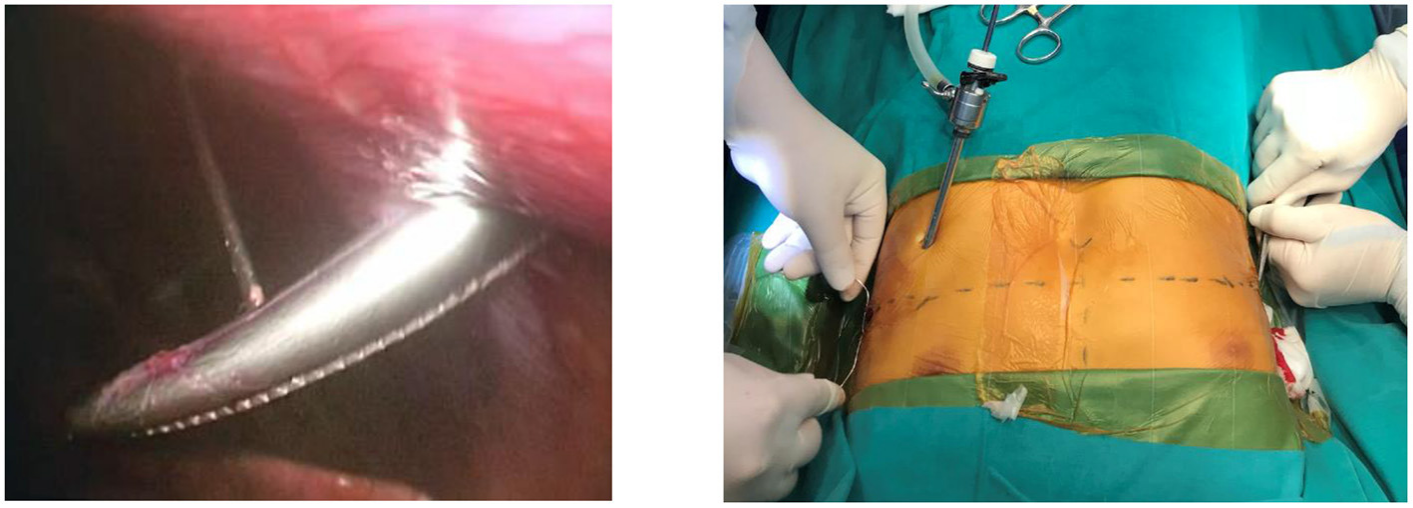
Pectus bar was anchored into the position by steel thread.

The steel thread was sewn around the ribs and was then tied to the top of the metal bar. Steel sutures on the one side combined with Vicryl sutures on the other were commonly utilized to fix the metal corrective bar. Bilateral steel sutures were used in severe PE patients with high risk of postoperative displacement of the steel support bar (Figure 4). The process of sewing steel threads was very safe under the observation of endoscopy. We did not use screw braces to fix the metal bar like the original Nuss surgery. Finally, the anesthesiologist squeezed the balloon through the endotracheal tube to allow the expanded lungs while gradually withdrawing the trocar and removing air from bilateral pleural space. Pleural drainage catheter was not placed.

### Statistical Analysis

A visual inspection was first performed on all data for coding errors, outliers, or funky distributions. No imputation was made for missing data. Continuous variables were expressed as mean, standard deviation (SD) with interquartile ranges. Categorical variables were presented as counts with percentage. *t* Test, Mann–Whitney *U* test, χ^2^ test, and Fisher exact test were applied to examine the differences between patients with bar removal and patients without bar removal. *p* < 0.05 was considered statistically significant. Data were processed using Stata 15 (StataCorp LLC, USA) for Windows.

### Ethical Approval

All procedures performed in studies involving human participants were in accordance with the ethical standards of the institutional and/or national research committee and with the 1964 Helsinki Declaration and its later amendments or comparable ethical standards. Informed consents were obtained from all individual participants included in the study. If the participants are younger than 18 years, or otherwise legally or medically unable to provide written informed consent, then consent is obtained from their parents or guardian. This study was approved by the Ethics Board of the Hanoi Medical University (no. 01NCS17/HMU IRB).

## Results

### Preoperative Clinical Characteristics of the Patients

Of 365 PE patients, the median age at operation was 15.61 ± 3.73 years (range = 5–27 years), most being child and adolescent. Three hundred nine patients (84.65%) were male. PE was commonly detected at puberty (*n* = 328, 89.9%); 84.4% of the patients were underweight [body mass index (BMI) <18.5 kg/m^2^], and no patient was recorded as overweight and obese. There were 1.37% of the patients who experienced previous PE repair surgery (*n* = 5). Among 365 patients, 288 patients had symmetric PE (78.90%), and 77 patients had asymmetric PE (21.10%) (Table 1). The numeric distribution of each morphologic type and subtype of pectus is described in Table 1.

**Table 1:** Preoperative clinical characteristics of patients.

**Characteristics**	**Patients (*N* = 365)**
Age	
Mean ± SD, years	15.61 ± 3.73
Range, years	5–27
Male sex, no. (%)	309 (84.65)
BMI, no. (%)	
Underweight (BMI < 18.5 kg/m^2^)	308 (84.38)
Normal (18.5 ≤ BMI < 23.0 kg/m^2^)	57 (15.62)
Overweight and obesity (BMI ≥23 kg/m^2^)	0 (0.00)
Time PE was detected, no. (%)	
At birth	11 (3.01%)
Childhood	26 (7.12%)
Puberty	328 (89.87%)
Previous PE repair surgery, no. (%)	5 (1.37)
Family history of PE, no. (%)	46 (12.60)
Comorbidity, no. (%)	
Prolonged respiratory tract inflammation	2 (0.55)
Asthma	1 (0.27)
Congenital heart disease	22 (6.03)
Scoliosis	1 (0.27)
Dementia	3 (0.82)
Marfan syndrome	1 (0.27)
PE defect type, no. (%)	
Bilateral symmetry	288 (78.90)
Asymmetry to the right	70 (19.18)
Asymmetry to the left	7 (1.92)
Morphologic classification ^a^, no. (%)	
Type 1A: prototype	252 (69.04)
Type 1B: broad–flat	70 (19.18)
Type 2A: eccentric	
Type 2A1: focal	14 (3.84)
Type 2A2: broad–flat	3 (0.82)
Type 2A3: long canal	1 (0.27)
Type 2B: unbalanced	24 (6.58)
Type 2C: combined	1 (0.27)
Clinical symptoms	
Inferiority complex, no. (%)	262 (71.78)
Chest pain while exercising, no. (%)	149 (40.82)
Dyspnea on exertion, no. (%)	218 (59.73)
Shortness of breath while exercising, no. (%)	203 (55.62)
Palpitations, no. (%)	298 (81.64)
Development of poor physical health, no. (%)	192 (52.60)

a *Morphologic classification by Park et al. (15)*.

Palpitations were present in 81.64% of the patients (*n* = 298). Other common symptoms were inferiority complex (*n* = 262, 71.78%), dyspnea on exertion (*n* = 218, 59.73%), shortness of breath while exercising (*n* = 203, 55.62%), development of poor physical health (*n* = 192, 52.60%), and chest pain while exercising (*n* = 149, 40.82%) (Table 1).

### Preoperative Subclinical Characteristics of the Patients

ECG abnormalities were documented in 41.10% of the patients (*n* = 150); the most common abnormality on ECG was sinus tachycardia (*n* = 90, 26.66%); increased right ventricular mass was present in 9.04% of the patients (*n* = 33), right bundle-branch block in 4.93% (*n* = 18), left bundle-branch block in 1.10% (*n* = 4), ectopic rhythm in 1.37% (*n* = 5), sinus bradycardia in 0.27% (*n* = 1), increased left ventricular mass in 0.27% (*n* = 1), and atrial fibrillation in 0.27% (*n* = 1). On Doppler echocardiography, 6.58% of patients had tricuspid valve regurgitation (*n* = 24), whereas the figure for mitral valve regurgitation was 3.84% (*n* = 14); patent foramen ovale was detected among 22 patients (6.03%); elevated pulmonary artery pressure was recorded in 33 patients (3.94%). Mean ejection fraction (EF) measured at 67.39 ± 5.91%. In 365 patients with PE presenting for surgical treatment, the mean forced vital capacity (FVC) was 63.80 ± 11.26, the mean forced expiratory volume in 1 second (FEV_1_) was 70.69 ± 11.8%, the mean FEV_1_/FVC was 111.91 ± 9.45, and the mean forced expiratory flow at 25–75% of the vital capacity (FEF_25%−75%_) was 98.62 ± 23.65. The mean Haller index was 3.81 ± 0.78 as measuring on chest CT scan and 3.83 ± 0.98 as measured on chest X-ray (Table 2).

**Table 2:** Preoperative subclinical characteristics of patients.

**Characteristics**	**Patients (*N* = 365)**
ECG abnormalities, no. (%)	150 (41.10)
Sinus tachycardia, no. (%)	90 (24.66)
Sinus bradycardia, no. (%)	1 (0.27)
Right bundle-branch block, no. (%)	18 (4.93)
Left bundle-branch block, no. (%)	4 (1.10)
Increased right ventricular mass, no. (%)	33 (9.04)
Increased left ventricular mass, no. (%)	1 (0.27)
Ectopic rhythm, no. (%)	5 (1.37)
Atrial fibrillation, no. (%)	1 (0.27)
Doppler echocardiography results	
Tricuspid valve regurgitation, no. (%)	24 (6.58)
Mitral valve regurgitation, no. (%)	14 (3.84)
Patent foramen ovale, no. (%)	22 (6.03)
Elevated pulmonary artery pressure, no. (%)	33 (9.04)
EF	
Mean ± SD, %	67.39 ± 5.91
Range, %	56–79.51
Pulmonary function tests	
FVC, mean ± SD	63.80 ± 11.26
FEV_1_%, mean ± SD	70.69 ± 11.80
FEV_1_/FVC, mean ± SD	111.91 ± 9.45
FEF_25%−75%_, mean ± SD	98.62 ± 23.65
Classification of pulmonary function test value	
FVC <80%, no. (%)	334 (93.56%)
FEV_1_ <80%, no. (%)	277 (77.59%)
FEV_1_/FVC <70%, no. (%)	2 (0.57%)
FEF_25%−75%_ <60%, no. (%)	12 (3.34%)
Chest CT scan	
Twisting the sternum, no. (%)	79 (21.64)
Scoliosis, no. (%)	3 (0.82)
Heart compression, the heart pushes to the left, no. (%)	58 (15.89)
Bronchiectasis, no. (%)	2 (0.55)
Haller index measuring on X-ray and CT scan	
Haller index on chest CT scan	
Mean ± SD	3.81 ± 0.78
Range	2.58–14.50
Haller index on chest X-ray	
Mean ± SD	3.83 ± 0.98
Range	2.56–14.50

*CT, computed tomography*.

### Operative Characteristics of the Patients

One bar was indicated for most patients (*n* = 350, 95.89%), and two bars were required in 15 patients (4.11%). The mean operative time (from cutting the skin to finishing the skin suture) was 49.54 min; 85.48% of the patients were carried through the left operative wound from the left to the right pleural cavity (*n* = 312), whereas 14.52% were carried from the right to the left pleural cavity (*n* = 53). Steel sutures on the left side combined with Vicryl sutures on the right were commonly utilized to fix metal corrective bar (*n* = 320, 87.67%). The epidural anesthesia was used to postoperative pain relief in 278 patients (76.16%). Intraoperative bleeding occurred in one case (Table 3).

**Table 3:** Operative characteristics of patients.

**Characteristics**	**Patients (*N* = 365)**
No. of bars (%)	
One bar	350 (95.89)
Two bars	15 (4.11)
VATS side, no. (%)	
Left VATS	308 (84.38)
Right VATS	43 (11.78)
Bilateral VATS	14 (3.84)
The steel introducer, no. (%)	
Left-to-right	312 (85.48)
Right-to-left	53 (14.52)
Method of metal corrective bar fixation, no. (%)	
Steel sutures (left) + Vicryl sutures (right)	320 (87.67)
Steel sutures (right) + Vicryl sutures (left)	34 (9.32)
Bilateral steel sutures	10 (2.74)
Bilateral Vicryl sutures	1 (0.27)
Postoperative pain relief by epidural anesthesia, no. (%)	278 (76.16)
Accidents in surgery, no. (%)	1 (0.27)
Operative time^a^	
Mean ± SD, min	49.54 ± 15.89
Range, min	20–140

a *Duration time starts from cutting the skin to finishing the suture and closure of the surgery site*.

### Postoperative Characteristics of the Patients

The length of postoperative hospitalization ranged from 1 to 13 days (mean = 5.1 days). Early postoperative complications were considered to be up to 30 days during hospital stay. The most common was pneumothorax (*n* = 5, 1.37%). Other events largely consisted of pleural bleeding/pleural fluid (*n* = 2, 0.55%), pleural hematoma (*n* = 1, 0.27%), pneumonia (*n* = 1, 0.27%), surgical wound infection (*n* = 1, 0.27%), incision fluid accumulation (*n* = 3, 0.82%), metal bar infection (*n* = 1, 0.27%), atelectasis (*n* = 3, 0.82%), and fever (*n* = 8, 2.19%). In addition, surgical wound infection (*n* = 2, 0.55%), metal bar deviation (*n* = 5, 1.37%), metal bar allergy (*n* = 10, 2.74%), recurrent PE (*n* = 2, 0.55%), and persistent PE (*n* = 5, 1.37%) were late postoperative complications. No deaths occurred in the present cohort (Table 4).

**Table 4:** Postoperative characteristics of patients.

**Characteristics**	**Patients (*N* = 365)**
Length of postoperative hospitalization	
Mean ± SD, days	5.10 ± 1.64
Range, days	1–13
Early postoperative complications	
Pneumothorax, no. (%)	5 (1.37)
Pleural bleeding/pleural fluid, no. (%)	2 (0.55)
Pleural hematoma, no. (%)	1 (0,27)
Pneumonia, no. (%)	1 (0.27)
Surgical wound infection, no. (%)	1 (0.27)
Incision fluid accumulation, no. (%)	3 (0.82)
Metal bar infection, no. (%)	1 (0.27)
Atelectasis, no. (%)	3 (0.82)
Fever, no. (%)	8 (2.19)
Late postoperative complications	
Surgical wound infection, no. (%)	2 (0.55)
Metal bar deviation, no. (%)	5 (1.37)
Metal bar allergy, no. (%)	10 (2.74)
Recurrent PE, no. (%)	2 (0.55)
Persistent PE, no. (%)	5 (1.37)
	**Patients (*****n*** **=** **175)**
Time the bar was *in situ*, no. (%)	
<1 years	3 (1.71)
≤ 1 to <2 years	16 (9.14)
≤ 2 to <3 years	103 (58.86)
≤ 3 to <4 years	49 (28.00)
≥4 years	4 (2.29)
Mean ± SD, months	28.89 ± 7.48
Range, months	2–49
Operative time at bar removal	
Mean ± SD, min	34.09 ± 10.61
Range, min	20–110
Length of hospitalization following bar removal	
Mean ± SD, days	2.40 ± 1.34
Range, days	1–14
Complications at bar removal	
Pneumothorax, no. (%)	19 (10.85)
Surgical wound infection, no. (%)	1 (0.57)
Incision fluid accumulation, no. (%)	1 (0.57)

### Bar Removal After the VATS-NUSS

Table 4 also indicated the outcomes related to the bar removal that was carried out in 175 patients (47.95%). The time the bar was *in situ* ranged from 2 to 49 months (mean = 28.89 ± 7.48 months). In three patients (1.71%), bar removal was performed early, <1 year, because of metal bar allergy. We recorded the mean operative time at bar removal of 34.09 ± 10.61 min and the length of hospitalization following bar removal of 2.4 ± 1.34 days. Pneumothorax, the most frequent complication, was diagnosed in 19 patients (10.85%). Besides, surgical wound infection in one patient and incision fluid accumulation in one patient were documented following bar removal.

### Midterm to Long-Term Outcome of Patients Undergoing VATS-NUSS

One hundred twenty-four of 365 patients (33.97%) were documented with midterm results at 6–30 months postoperatively. Of 124 patients, most patients had unremoved bar (*n* = 121, 97.58%), whereas only three patients had the bar removed (2.42%). Most patients had body weight gain (*n* = 97, 78.23%) and increased physical activity and improved health (*n* = 115, 92.74%) in the postoperative midterm period. The mean BMI index was 18.77 ± 0.92 kg/m^2^, and the Haller index on chest X-ray was 2.44 ± 0.15. Postoperative long-term results over 30 months were also assessed with 220 (60.27%) of 365 patients (Table 5). Of these, 172 received bar removal (78.18%), and 48 had not yet received bar removal (21.82%). Two hundred seventeen patients (98.64%) had both body weight gain and increased physical activity and improved health. The mean BMI index was 19.02 ± 0.99 kg/m^2^, and the Haller index on chest X-ray was 2.45 ± 0.21.

**Table 5:** Midterm to long-term postoperative outcome in PE patients.

**Midterm postoperative outcome (*****n*** **=** **124)** **(6–30 months postoperatively)**				
	**All patients (*n* = 124)**	**Bar removal**		***p*-value**
		**Yes (*n* = 3)**	**No (*n* = 121)**	
	**Count (% of total)**	**Count (% of total)**	**Count (% of total)**	
Body weight gain	97 (78.23)	3 (100.00)	94 (77.69)	1.000^*^
Increased physical activity and improved health^a^	115 (92.74)	3 (100.00)	112 (92.56)	<0.05^*^
	**Mean (SD)**	**Mean (SD)**	**Mean (SD)**	
BMI index, kg/m^2^	18.77 ± 0.92	20.30 ± 1.11	18.73 ± 0.89	<0.05^**^
Haller index on chest X-ray	2.44 ± 0.15	2.5 ± 0.17	2.44 ± 0.15	0.382^**^
**Long-term postoperative outcome (*****n*** **=** **220)** **(over 30 months postoperatively)**				
	**All patients (*****n*** **=** **220)**	**Bar removal**		***p-*****value**
		**Yes (*****n*** **=** **172)**	**No (*****n*** **=** **48)**	
	**Count (% of total)**	**Count (% of total)**	**Count (% of total)**	
Weight gain	217 (98.64)	171 (99.42)	46 (95.83)	0.321^*^
Increased physical activity and improved health^a^	217 (98.64)	171 (99.42)	46 (95.83)	0.990^*^
	**Mean (SD)**	**Mean (SD)**	**Mean (SD)**	
BMI index, kg/m^2^	19.02 ± 0.99	19.13 ± 1.01	18.62 ± 0.82	<0.001^**^
Haller index on chest X-ray	2.45 ± 0.21	2.46 ± 0.23	2.41 ± 0.14	0.067^**^

* *χ^2^ Test*.

** *Mann–Whitney U test*.

a *Assessed through face-to-face interview with patient or their parents by primary care physician*. *p-values with statistically significance are given in bold*.

## Discussion

In the emerging clinical practice in a developing country such as Vietnam, it difficult for us to acquire all important data for a large cohort of consecutive PE patients who underwent VATS for the NUSS procedure. Hence, from the Vietnamese surgeons' initial experience, we believe that the results from this cohort enable to give a real condition in clinical practice valuably not only for Vietnam but also for other countries having similar resource-scarce settings. In Vietnam, the cost and insurance coverage for the PE treatment have not been the main barriers for the patients and their family. The main issue in our condition is that there is no primary PE screening system at postpartum, evident in the late detection of PE. Although the number of children with PE that was detected at an early stage increased within the recent 5 years in Vietnam's large surgical institutions, many barriers to timely referral exist in remote rural and mountainous areas. With the ongoing development in other developed countries, many charity organizations across the world have come to Vietnam to lend a helping hand in the screening and treatment of PE; however, that concern is not enough to have a general picture in the detection and treatment of PE in Vietnam. To date, several ethnic minority communities lack adequate knowledge about PE in children and its lesions, with our primary healthcare system lacking good communication for birth defects between hospitals and the community. The mean age in our study is 15.61 ± 3.73 years (range = 5–27 years). When dividing the age according to the school-age groups of Vietnamese children, we found that the highest percentage of children undergoing VATS for the NUSS procedure were in 12- to 15-year age group (41.64%), which was known as the child's puberty period. In our institution, usually, the family takes the child to the hospital for examination and treatment when the child has evidently presented PE symptoms. Noticeably, 48.23% was in the age of 16 years or older, partially showing that the families' understanding and awareness of PE are still limited in Vietnam, and also gaps exist in the national surveillance and public health surveillance systems of congenital anomalies. As known from the literature, proper metal bar shape is crucial in NUSS for PE patients. Kelly et al. favored a gentle semicircular curve laterally with a 2- to 4-cm flat segment in the center to support the sternum (16). Slight overcorrection of the depression is to be preferred to undercorrection because the authors believed it minimizes the risk of recurrence and decreases the risk of buckling of the cartilages (16). Park et al. favored an asymmetrically bent bar for patients with an asymmetric depression (17). In this cohort, we used a ruler bent to the desired thoracic shape and then bent the metal bar to the shape and size of the ruler. We began to bend the middle of the bar gradually to its ends; bars are usually taken to be 1 inch smaller compared to the distance between the two middle armpit lines. We have found that symmetrically bent bars tend to be stable on the patients' chest.

In our patient series, most patients received only one bar (95.89%), whereas 4.11% of the patients received two bars. We indicated the two-bar insertion for most elderly patients with severe PE and wide concave area. No case in our institution had three metal bars placed yet. Our finding was consistent with previous reports when both revealed that the number of implanted steel bars was greater with age (18–20). Compared to previous studies (19, 20), the number of patients receiving two metal bars was lower in our study. The patient's chest wall becomes more solid after puberty and harder to bend; hence, introducing more than one bar is done to prevent postoperative pain and pressure on the chest wall by preventing displacement of the bar. Previous authors suggest that the PE patients with Marfan syndrome or grand canyon PE and asymmetrical PE require two metal bars. In addition, the use of two metal bars during the first treatment also becomes the standard of minimally invasive surgery for PE in several reports. To date, not only does the rate of using two metal bars increase gradually in recent years, but also the insertion of three metal bars (21–23).

Previous reports have shown the safety of thoracoscopic NUSS, with decreased frequency of serious intraoperative and postoperative complications, especially in patients with complex PE, a previous history of intrathoracic surgery and recurrent PE (7, 9, 15). VATS technique was acquired for all PE patients in the current cohort, and CO_2_ insufflation was used to create an artificial pneumothorax with the aim of increasing the empty pleural space. In the early years of implementing VATS in NUSS in our institution, the surgeons stand to the right of the patient using the supportive thoracoscopic approach, consistent with the classic NUSS that chooses the entrance from the right chest wall. Then, the left thoracoscopy is mostly used during recent years (19, 24) because we find this surgical access in line with the doctors' experience in our institution. In particular, bilateral thoracoscopic approach was applied in 14 patients because of severe PE compressing the lungs and heart and failed Ravitch procedure. In the early years of implementing VATS in NUSS, we applied both thoracoscopic approaches including the right chest wall and the left chest wall. In our opinion, the surgical field of view in the left thoracoscopic approach is wide enough to clearly observe the entire pleural cavity, left lung, blood vessel, heart, pericardium, mediastinum, and diaphragm; hence, serious complications can be avoided. Several authors favored placing the trocar in the middle axillary line (11, 25). However, we found that the trocar placed in the middle axillary line can result in that the vision can be hindered by the heart, lungs, and diaphragm, especially in cases of asymmetrical severe PE. We agree with Hendrickson et al. and Palmer et al. that left thoracoscopy has the advantage over right thoracoscopy and does not increase the risk of heart injury (8, 10). Hemostasis is well-controlled, and lung damage is avoided under endoscopic observation. During the process of ballooning through the endotracheal tube to enlarge the bilateral lungs, we clearly observed that the two lungs were enlarged, and until the lungs were maximized, when the pleural space was empty, we withdrew the endoscopic trocar. Therefore, pleural drainage catheter was not placed in surgery.

During the surgery, no serious complications and no fatalities occurred in our series. Operative bleeding complication was observed in one case. This is the case of intercostal arterial injury in the position of attaching the steel bar to the rib on left side. Effective hemostasis is then controlled by thoracoscopy. The most frequent complication in the early postoperative period was pneumothorax. The occurrence of this complication varies widely between 1.3 and 64% in the literature (9, 26–29). In our patients, the pneumothorax resolved spontaneously and did not require additional surgical intervention. Besides, no patient with postoperative bleeding required a blood transfusion, and no other serious complications or death was recorded in our study. Late postoperative complications are events that occur when the patient is discharged from the hospital, such as metal bar deviation, metal bar allergy, or surgical wound infection. Bar displacement is one of the most common complications (30). We recorded a bar displacement rate of 1.37%, and no major bar displacement in all patients. According to early literature, the incidence of bar displacement was 3–20%, and this incidence is lower in our study (1.37%) (15, 17, 31). Our approach with the mechanism-based bar fixation techniques by case-by-case analysis seems to be an effective and safe method of bar fixation in minimally invasive PE repair. Metal bar allergy also occurred in 2.74% of our patients (*n* = 10) with their manifestations such as mild fever, inflammation, wound fluid buildup, blisters, and erythema. Our study had similar proportion of metal bar allergy compared to others such as that of Nuss and Kelly (32) with 2.9% and Kelly et al. (16) of 2.8%. In particular, we found that 6 of 10 patients with metal bar allergy had a history of allergic diseases such as allergic rhinitis, drug allergy, weather allergy, food allergy, and asthma. Here we queried only for history of allergic diseases prior to surgery. The test for metal allergy before the operation was not available in clinical condition at our institution; therefore, we were incapable of testing routinely those for metal allergy. This helps us gain more experience in the prognosis, diagnosis, and treatment of allergy drugs, as well as closely monitor allergy status after surgery for appropriate management measures.

NUSS is known as a prepared clean surgery. Although very few wound infections were reported, these are extremely serious because a foreign material has been placed inside the body. In our study, there were two patients with late wound infection (0.55%). They received prompt antibiotic treatment and were discharged after 7- to 10-day treatment. There were no cases of deep infection to metal bar. Some studies in the literature showed the incidence of late wound infection was 1.5% (33, 34). In addition, other complications should be also noted in our report. Five patients had chest wall pain lasting 1 and 2 months postoperatively, for which oral pain relievers were taken and breathing exercises were performed; their pain then subsided and disappeared. There were two cases of recurrent PE and five cases of persistent PE after bar removal operation.

Midterm to long-term postoperative outcomes in PE patients were also assessed in the present cohort. Most patients had body weight gain after surgery. Through information self-reported by patients or their family, we also found increased physical activity and improved health. The Haller index, which is the ratio of the widest transverse (T) diameter of the chest to the shortest distance between the anterior spine and posterior sternum, has been used to quantify the severity of PE. Also, the size of the PE was postoperatively evaluated according to the Haller index based on two projection chest X-rays—posteroanterior and right lateral. We measured the mean Haller index of 2.44 ± 0.15 at 6 to 30 months postoperatively, while the Haller index on chest X-ray was 2.45 ± 0.21 over 30 months. In our patient cohort, bar removal was indicated based on the appropriate duration of bar maintenance, removal techniques, and strategies to avoid complications. For children up to 12 years old, the bar was maintained for 2 years, whereas in 12- to 20-year-old individuals, it was maintained for 2.5 years, and that in adults older than 20 years was maintained for 3 years.

For the first time in Vietnam, we conducted a large retrospective cohort of consecutive patients with the diagnosis of PE undergoing VATS for the NUSS procedure at a major academic Medical Center (Viet Duc University Hospital). Therefore, our study should be viewed in light of its main limitations. The study's retrospective nature and being a single-center study need be acknowledged. Because this was a retrospective review of medical records, the follow-up period data from the patients' medical records in our institution were available only for 124 of 365 patients at 6 to 30 months postoperatively and 220 of 365 patients at 30 months postoperatively. Our study with relative sample size and from one institution needs to be highlighted, potentially showing a limited validity and not enabling to represent those of other centers. In addition, because of the unavailability of retrospective medical records, we were unable to provide data about PE recurrence after the removal of metal bars as well as midterm and long-term postoperative pain. Because of the scope of study objectives, it is interesting for the authors to, in detail, evaluate the different postoperative time points and surgical outcomes, as well as to establish prognostic models for postoperative results in a further study. In particular, the metal support is still in place in a number of PE patients, not enabling us to have complete evaluation of postoperative results in the present study.

## Conclusions

The VATS technique in NUSS for PE was a safe and effective operation, with minimizing the occurrence of serious intraoperative and postoperative complications. From our initial one-institution experience in a lower-middle income country, favorable midterm to long-term postoperative outcomes in PE patients were obtained with VATS treatment using the minimally invasive Nuss method. Current rare evidence drawn from this cohort enables to give a real picture on the application, modification, and development of VATS in NUSS not only for Vietnam but also for other countries having similar resource-scarce conditions.

## Data Availability Statement

The original contributions generated for this study are included in the article/supplementary materials, further inquiries can be directed to the corresponding author/s.

## Ethics Statement

The studies involving human participants were reviewed and approved by the Ethics Board of the Hanoi Medical University (No. 01NCS17/HMU IRB). Informed consents were obtained from all individual participants included in the study. If the participants are under the age of 18, or otherwise legally or medically unable to provide written informed consent, then consent is obtained from their parents or guardian.

## Author Contributions

T-MN and Q-HD: conceptualization and writing—original draft preparation. T-MN, V-TL, H-UN, and Q-HD: methodology, validation, and resources. N-KD, H-LV, and K-DV: software. H-LV and K-DV: formal analysis. T-MN, H-UN, H-SP, N-TV, V-AN, and Q-HD: investigation and visualization. Q-HD: data curation and supervision. T-MN, H-UN, H-SP, N-TV, V-AN, H-LV, and Q-HD: writing—review and editing. N-KD and K-DV: project administration. T-MN: funding acquisition. All authors have read and agreed to the submitted version of the manuscript.

## Conflict of Interest

The authors declare that the research was conducted in the absence of any commercial or financial relationships that could be construed as a potential conflict of interest.
